# A multiple-trait analysis of ecohydrological acclimatisation in a dryland phreatophytic shrub

**DOI:** 10.1007/s00442-021-04993-w

**Published:** 2021-07-31

**Authors:** M. Trinidad Torres-García, María J. Salinas-Bonillo, Jamie R. Cleverly, Juan Gisbert, Manuel Pacheco-Romero, Javier Cabello

**Affiliations:** 1 Department of Biology and Geology, University of Almería, Carretera de Sacramento s.n, La Cañada de San Urbano, 04120 Almería, Spain; 2 Andalusian Center for the Monitoring and Assessment of Global Change (CAESCG), University of Almería, Almería, Spain; 3 School of Life Sciences, Faculty of Science, University of Technology Sydney, PO Box 123, Broadway, NSW 2007 Australia

**Keywords:** Depth-to-groundwater gradient, Ecophysiological threshold, Groundwater salinity, Plant functional traits, Rhamnaceae, *Ziziphus lotus*

## Abstract

Water is the main limiting factor for groundwater-dependent ecosystems (GDEs) in drylands. Predicted climate change (precipitation reductions and temperature increases) and anthropogenic activities such as groundwater drawdown jeopardise the functioning of these ecosystems, presenting new challenges for their management. We developed a trait-based analysis to examine the spatiotemporal variability in the ecophysiology of *Ziziphus lotus*, a long-lived phreatophyte that dominates one of the few terrestrial GDEs of semiarid regions in Europe. We assessed morpho-functional traits and stem water potential along a naturally occurring gradient of depth-to-groundwater (DTGW, 2–25 m) in a coastal aquifer, and throughout the species-growing season. Increasing DTGW and salinity negatively affected photosynthetic and transpiration rates, increasing plant water stress (lower predawn and midday water potential), and positively affected Huber value (sapwood cross-sectional area per leaf area), reducing leaf area and likely, plant hydraulic demand. However, the species showed greater salt-tolerance at shallow depths. Despite groundwater characteristics, higher atmospheric evaporative demand in the study area, which occurred in summer, fostered higher transpiration rates and water stress, and promoted carbon assimilation and water loss more intensively at shallow water tables. This multiple-trait analysis allowed us to identify plant ecophysiological thresholds related to the increase in salinity, but mostly in DTGW (13 m), and in the evaporative demand during the growing season. These findings highlight the existence of tipping points in the functioning of a long-lived phreatophyte in drylands and can contribute to the sustainable management of GDEs in southern Europe, paving the way for further studies on phreatophytic species.

**Supplementary Information:**

The online version contains supplementary material available at 10.1007/s00442-021-04993-w.

## Introduction

Water is an essential global resource for humans and ecosystems, particularly in arid regions where it is the most limiting factor (Newman et al. 2006). Arid and semiarid regions are characterised by low and shifting water availability across space and time (Eamus et al. 2013), thus vegetation has to live with water limitation or explore new water sources below ground (Arndt et al. 2001; Nardini et al. 2014). In this sense, groundwater reservoirs are crucial for the functioning of vegetation (O’Grady et al. 2006) in the ecosystems that have access to this hidden water source, the so-called groundwater-dependent ecosystems (GDEs) (Eamus et al. 2006). GDEs of arid regions are highly vulnerable to alterations in the hydrological regime, because their structure and functioning depend on it (Eamus et al. 2006). Groundwater condition, i.e. water quality and quantity, affects GDEs, and groundwater exploitation or pollution jeopardises their structure and function as well as the species that constitute them (Zolfaghar et al. 2014; Eamus et al. 2015). How the function of GDEs in drylands is affected by groundwater variations is a primary concern for scientists, managers and policymakers who have to design sustainable plans to manage groundwater resources in the face of climate change (Kløve et al. 2014).

Fluctuations in groundwater depth can be detrimental to the functioning of GDEs and the deep-rooted phreatophytic vegetation that tap groundwater (Naumburg et al. 2005). Groundwater drawdown can salinize both soils and water in arid regions due to the exclusion of salts by plants during water uptake or to the exposure of deeper and saltier groundwater (Jobbágy and Jackson 2007; Runyan and D’Odorico 2010). Seawater intrusion, as an indirect effect of water table decline near the coast, is one of the main drivers of coastal aquifer salinization. Likewise, groundwater availability for plants can depend on salinity, which has shown substantial consequences in phreatophytic productivity, even inducing diebacks (Jolly et al. 1993; Doody and Overton 2009; Runyan and D’Odorico 2010). Even though salinity is a significant abiotic stress that intensifies drought impacts and water unavailability, there is little research on plant response to both groundwater salinity and depth (Kath et al. 2015; Hussain and Al-Dakheel 2018).

Groundwater-dependent ecosystems are amongst the terrestrial ecosystems most vulnerable to climate change effects, and their ability to persist will depend on the resilience of phreatophytic vegetation to groundwater decline (Hultine et al. 2020). It is widely recognised that anthropogenic activities alter the groundwater regime, either directly through groundwater exploitation or indirectly through land-use change (Eamus et al. 2015, 2016), which in turn can promote soil and groundwater salinization (Jobbágy and Jackson 2007; Nosetto et al. 2008). In addition, future climate change, expressed in the Mediterranean basin by a reduction in precipitation and an increase in temperature (Giorgi and Lionello 2008), will reduce groundwater recharge and raise evapotranspiration rates. Modelling carbon–water relationships will help us predict how hydrological changes can affect GDEs in terms of survival and productivity, thus addressing human impacts (Naumburg et al. 2005; Newman et al. 2006). To test vegetation response to altered water regimes, scientists usually resort to spatial gradients of aridity, altitude, water availability, and soil nutrients, amongst others (Lavorel and Garnier 2002; Wright et al. 2004; Mitchell and O’Grady 2015). Topography, for instance, can promote gradients in water availability, which cause critical variations in plant structure and function (Williams et al. 1996). The study of a species response to reduced water availability along environmental gradients will provide insight for identifying ecophysiological thresholds in phreatophytic vegetation (Eamus et al. 2006). Such thresholds might be related to the limits for maintaining high ecophysiological functioning in a “safe operating space” rather than the physical disconnection between vegetation and groundwater. Despite the definition of these tipping points is still scarce, particularly in European GDEs (Froend and Drake 2006; González et al. 2012; Garrido et al. 2016), its knowledge is essential for a sustainable management in drylands.

Plant functional traits that refer to morphological, physiological, and phenological characteristics of the vegetation (Perez-Harguindeguy et al. 2013) provide insight about plant ecological strategies, contributing to understanding how vegetation responds to abiotic factors (Lavorel and Garnier 2002). This “bottom-up” approach that relates plant traits to environmental gradients is a way forward for facing important ecological questions (Cornelissen et al. 2003). In GDEs, plant functional traits are the vehicle to assess different aspects of ecosystem functioning as they respond to changes in the hydrologic regime (Eamus et al. 2006). In this sense, an understanding of the connection between morpho-functional and hydraulic traits with groundwater characteristics (i.e. groundwater depth, salinity, and temperature) will be crucial for predicting climate change effects upon GDEs.

Numerous morpho-functional traits such as Huber value (Hv), wood density, specific leaf area (SLA), and gas-exchange rates show variation across depth-to-groundwater (DTGW) gradients in arid and semiarid environments (Stromberg et al. 1996; Gazal et al. 2006; Butler et al. 2007; Carter and White 2009; Zolfaghar et al. 2014; Osuna et al. 2015; Sommer et al. 2016; Nolan et al. 2017a). Hydraulic traits such as water potential are strongly correlated with DTGW gradients, as shown in phreatophytic oaks, eucalyptus, and acacias from California and Western and Central Australia (Carter and White 2009; Osuna et al. 2015; Nolan et al. 2017a). Here, we explore a GDE dominated by the winter-deciduous phreatophyte *Ziziphus lotus* (L.) Lam. (*Rhamnaceae*) in a small coastal plain in the southeast of Spain where spatiotemporal variations in groundwater salinity and temperature were also assessed. We evaluated the relationships amongst a broad suite of traits including stem water potential, gas-exchange rate, intrinsic water-use efficiency (WUEi), Huber value (Hv), wood density, and specific leaf area (SLA), across a naturally occurring DTGW gradient related to distance from the coastline. We also assumed that seawater intrusion could more adversely affect plants near the coast. Thus, we hypothesised that spatiotemporal fluctuations of both groundwater availability and quality would drive differences in the ecophysiological functioning of *Z. lotus*. These differences could help us to identify ecophysiological thresholds, which will provide valuable insight to face upcoming management challenges in GDEs. To test these hypotheses, we address the following specific questions: Are there spatiotemporal variations in plant functional traits? Do these variations respond to groundwater conditions? Is there any discernible threshold in the ecophysiological functioning of *Z. lotus?* What factors drive the threshold?

## Methodology

### Site description

**Fig. 1:**
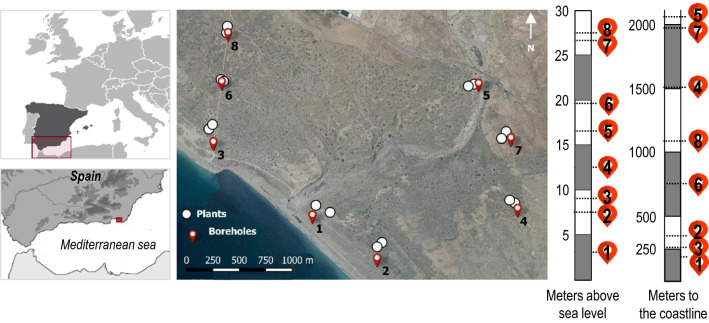
Location of the study area in the coastal plain of Cabo de Gata-Níjar Natural Park, southeastern Spain. Distribution of the boreholes (1 to 8) and the related plants of *Ziziphus lotus* (circles, *n* = 16) are shown. Bars indicate distance to the coastline (*m*) and metres above sea level (m.a.s.l) at each site

The study was conducted on a coastal plain at the western part of the Cabo de Gata-Níjar Natural Park, southeastern Spain (Fig. 1). The climate is characterised as Mediterranean and semiarid, with hot and dry summers and mild, wet winters. Mean annual temperature is 18 ºC, and mean annual precipitation is 200 mm (Machado et al. 2011), which is unevenly distributed during spring and autumn in scarce, short, and infrequent rainfall events (Online Resource 1). The coastal plain is underlain by a shallow aquifer, comprised of Plio-Pleistocene conglomerates, with aeolian sands beneath it and Pliocene marine marls at the base. The geology originated from the sedimentary fill of the Bay of Almería with materials from the Sierra Alhamilla mountains (1000 m.a.s.l) and from coastal marine deposits from the Quaternary period (Vallejos et al. 2018). Eight boreholes located along the study area form a net for groundwater observation that discerns between 3 sites (east plain, west plain, and the seasonal stream that crosses it) and shows a natural occurring DTGW gradient based on coastline distance and topography.

The winter-deciduous phreatophyte *Z. lotus* is the dominant species of this coastal plain ecosystem, which is comprised of *Z. lotus* and other shallow-rooted Mediterranean shrubs such as *Lycium intricatum*, *Salsola oppositifolia,* and *Withania frutescens* (Tirado 2009). *Z. lotus* distributes along the Mediterranean basin, being native from North Africa, the Middle East, and southern Europe, mainly Spain, where it constitutes one of the few terrestrial GDEs in European drylands (Guirado et al. 2018; Torres-García et al. 2021*).* It is a slow-growing, long-lived shrub that has not substantially changed in size or shape in the past 70 years in the study area. The vegetation on this coastal plain shows a patchy, dispersed pattern typical of arid and semiarid Mediterranean regions, where *Z. lotus* is associated with biodiversity islands (Tirado 2009). *Z. lotus* is responsible for most of the photosynthetic activity during summer, whereas the rest of the vegetation constituting the island grows in winter, entailing a replacement in the drivers of the primary productivity of the ecosystem (Guirado et al. 2018). *Z. lotus* partially depends on groundwater to survive (Torres-García et al. 2021) by developing a dual root system that can reach up to 60 m deep (Le Houérou 2006) whilst also maintaining active roots near the surface. Vegetation sampling was made on a total of 16 adult individuals of *Z. lotus* (1–3 m tall and 50–200 m^2^ area) selected next to each bore (two per bore at a maximum distance of 130 m) (Fig. 1) in three specific periods of 2019 growing season: late-spring (May), mid-summer (July), and late-summer (September).

### Hydrologic and climatic measurements

Each bore contained two sensors (Hobo U20 Water level logger and Hobo U24 conductivity logger, Onset Comp. Coorp., Bourne, MA, USA) to obtain DTGW, electrical conductivity (i.e. salinity), and groundwater temperature (*T*_GW_) every 15 min since May 2019. For regression analysis, we obtained mean values from each of the sampling periods. In the same way, we collected daily climatic data from Almería airport meteorological station (Spanish meteorological agency) located 8 km from the study area. Monthly precipitation (P) and mean monthly temperature (*T*_air_) were used (measured with a Thies Precipitation Transmitter, Göttingen, Germany; and a Vaisala HUMICAP HMP155, Helsinki, Finland, respectively).

### Plant traits

We analysed three traits related to the plant water potential, four physiological traits from leaf gas-exchange rates, and three morphological traits We measured water potential during the growing season at predawn (Ψ_pd_) and midday (Ψ_md_) in four stems on each of the 16 individuals using a Scholander pressure chamber (SKPM1405, Skye Instruments, Powys, UK). Measurements were taken before sunrise for Ψ_pd_ (from 06:00 to 07:00 h in May and July and from 06:30 to 07:30 h in September) and during the peak insolation for Ψ_md_ (between 13:00 and 14:00 h). Mean values for each plant and period were calculated, and the maximum daily range (ΔΨ_max_) was derived afterwards as the difference between Ψ_pd_ and Ψ_md_. We measured leaf gas exchange in 8 sun-exposed leaves per plant around four different points of the outer part of the canopy (north, east, south, and west) between 10:00 and 13:00 h on the same days as water potential was measured. A portable infrared gas analyser (Li-6400XT; LI-COR Inc., Lincoln, NE, USA) was used with the following conditions in the chamber to standardise all measures: flow rate, 400 µmol s^−1^; CO_2_ concentration, 400 µmol mol^−1^; and light intensity, 1800 µmol m^−2^ s ^−1^. Ambient temperature was kept, which varied between 25 and 30 ºC. We obtained photosynthetic rate (*A*), stomatal conductance (*g*_s_), transpiration rate (*E*), vapour pressure deficit (VPD), and WUEi was calculated from the ratio between *A* and *g*_s_.

Finally, to gather morphological traits, we cut three branches of similar size per plant in July from which all leaves were removed. We measured sapwood cross-sectional area with a digital calliper in the base of each branch. Sapwood was distinguished from heartwood by the colour difference. We also estimated wood density as the volume of a piece of branch (π × radius^2^ × length) divided by its dry weight (after 48 h at 60 ºC). We scanned all the leaves with a digital leaf area metre (WinDIAS, Cambridge, UK) to calculate total leaf area per branch and used ten of the leaves to estimate the SLA of the plants, which represents the relationship between the leaf area and its dry weight (after 48 h at 60 ºC). We calculated the Hv per plant from the ratio between the mean sapwood cross-sectional area to the mean total leaf area.

### Data analysis

We applied a two-way ANOVA for each groundwater characteristic and functional trait to assess intraspecific variability, both temporal (between sampling periods) and spatial (between sampling sites). Since SLA, Hv, and wood density were only measured once, we performed a one-way ANOVA for these traits. All traits were log-transformed except for water potentials due to the negative nature of their values. We undertook Tukey’s HSD post hoc test after significant differences were found. To further examine the effects of the main stressors (salinity and DTGW) on plant response, differences in gas exchange and water potential traits between pairs of bores were tested by a Student’s *t* test. We also performed multiple bivariate linear regressions to test whether a single regression could describe individual functioning. Some regressions were made with mean values, as variability over time was not observed, whereas others were made with monthly data to detect seasonal patterns. Finally, we analysed multiple-trait relationships across all variables with a principal component analysis (PCA). Traits were scaled prior to the analysis to obtain a unit variance. Spearman correlation analysis was applied, and the contribution of each trait in the PCA was assessed to select those variables that provide the best representation and improve the analysis. Because of that, SLA, WUEi, and wood density were not included in the final analysis. We performed all analyses in R 3.5.2 (R Core Team 2018).

## Results

### Spatiotemporal variations in groundwater

**Fig. 2:**
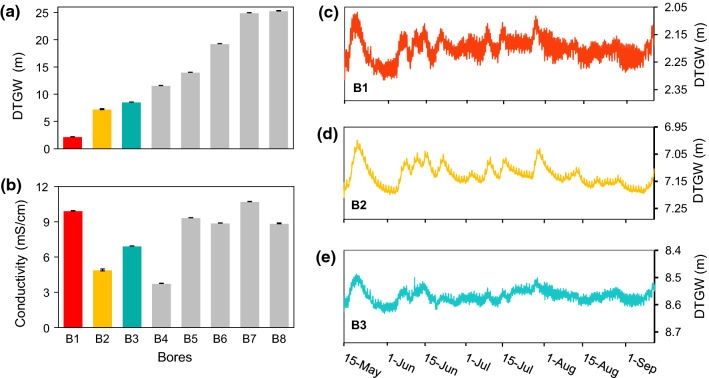
Mean **a** depth-to-groundwater (DTGW, m) and **b** groundwater salinity (electrical conductivity, mS/cm) at different sites (bores) ± SE. Temporal fluctuations of DTGW in the shallowest and closest to the coast bores are also shown (**c**, **d**, and **e**)

We observed significant differences in DTGW, salinity, and T_GW_ between sites, across the growing season, and for their interaction (*P* < 0.001; *df* = 7, 4). These variables increased during the growing season, although with different patterns. First, DTGW that ranged from 2.1 m (bore 1) to 25.4 m (bore 8) (Fig. 2a) increased across the growing season, although not substantially (Online Resource 2). It was just at the inner-plain sites where an average increase of 18 cm was observed at the end of the season (bore 8). Near the coast, we observed more noticeable temporal fluctuations although these did not entail overall DTGW increments (Fig. 2c, d, e, and Online Resource 2). Second, T_GW_ gradually increased during summer (Online Resource 3), despite its narrow range in average monthly values (from 21.78 to 23.98 °C, Online Resource 2). These rises mainly affected bores with the shallowest water tables such as bore 1, that showed wider fluctuations, and bore 2, that had the steepest increase. Finally, groundwater salinity, which ranged from 3360 µS/cm (bore 4) to 11000 µS/cm (bore 7), increased in bores 1, 3 and 7, but particularly in bore 7 where a rise in almost 1000 µS/cm was observed (Online Resource 2 and Online Resource 3). For these three groundwater properties, fluctuations were larger near the coast where water tables were shallower (bores 1–3) than in the other bores.

### Spatiotemporal variations in plant traits and their relationship with groundwater

Plant traits also showed significant differences between sampling periods, sites, and the interaction between them (Table 1 and Online Resource 4). Overall, gas exchange (*A* and *E*) in *Z. lotus* leaves was higher in summer (July and September) and at those sites with the shallowest water tables. Regarding water loss, plants from bores 1–4 (DTGW < 11.6 m) showed the highest *g*_s_, especially during July and September when it reached 0.42 ± 0.03 mol H_2_O m^−2^ s^−1^, whereas bores 5–8 (DTGW > 14.0 m), had the lowest values. It is also noticeable that high rates of *E* for plants from bores 1, 2, and 3 occurred in July and September, but also from bore 8 (25.3 m). Nevertheless, *A* showed significant differences in summer just at some locations (interaction term, *P* < 0.001, *df* = 14), although general differences between months were not observed (individual term, *P* = 0.1, *df* = 2, Online Resource 4). Individuals next to bores 2 and 5 (with a DTGW of 7.3 and 14.0 m, respectively) had higher photosynthetic rates in July, whereas plants near bores 6 and 8 (with 19.3 and 25.3 m, respectively) showed lower values at the end of summer (Table 1). In general, individuals next to bores 1 and 2 had the highest rates of *A*, whereas bore 8 showed the lowest ones. Contrary to *A*, WUEi was low at not only the shallowest water tables, but also at the deepest and saltiest ones. Regarding water potential, more negative values of both Ψ_pd_ and Ψ_md_ were observed in July and September at sites with the highest DTGW (bores 5–8). Ψ_pd_ ranged between − 0.32 ± 0.02 MPa in May and − 1.55 ± 0.09 MPa in September (at bore 2 and bore 8, respectively), whereas Ψ_md_ showed values between − 1.18 ± 0.04 MPa in May and − 3.13 ± 0.10 MPa in July (bore 4 and bore 8, respectively). Hv also showed significant differences across sites (*P* = 0.027) (Online Resource 5). The Hv of the plants at bore 1 with shallow groundwater (3.58 ± 0.08) was significantly lower than that of plants at bores 7 and 8 with deep groundwater (11.40 ± 0.22 and 9.34 ± 0.84, respectively). Neither SLA nor wood density showed significant spatial variability.

**Table 1:** Summary of mean values of traits (± SE) from plants next to each bore in the three sampling periods: May, July, and September

Bore DTGW (m)	Month	A (µmol CO_2_ m^−2^ s^−1^)	g_s_ (mol H_2_O m^−2^ s^−1^)	E (mmol H_2_O m^−2^ s^−1^)	WUEi (µmol CO_2_ /mol H_2_O)	Ψ_pd_ (MPa)	Ψ_md_ (MPa)	VPD (kPa)
Bore 1	May	15.32 ± 1.69 a	0.33 ± 0.05 a	7.84 ± 0.81 a	50.53 ± 3.88 a	− 0.42 ± 0.03 a	− 1.74 ± 0.19 a	2.82 ± 0.04 a
2.2 m	July	13.77 ± 1.87 a	0.35 ± 0.04 a	10.94 ± 1.08 b	37.92 ± 2.54 a	− 0.63 ± 0.05 a	− 2.33 ± 0.17 b	3.39 ± 0.06 b
	Sep	15.51 ± 1.12 a	0.32 ± 0.02 a	12.16 ± 0.67 b	48.11 ± 1.95 a	− 1.08 ± 0.05 b	− 2.98 ± 0.08 c	3.91 ± 0.03 b
Bore 2	May	16.17 ± 1.77 a	0.23 ± 0.02 a	5.81 ± 0.48 a	73.84 ± 5.30 a	− 0.32 ± 0.02 a	− 1.23 ± 0.09 a	2.63 ± 0.04 a
7.3 m	July	24.88 ± 2.36 b	0.40 ± 0.05 b	10.60 ± 1.05 b	73.52 ± 8.85 a	− 0.64 ± 0.04 b	− 2.49 ± 0.04 b	2.96 ± 0.06 a
	Sep	15.83 ± 1.47 a	0.32 ± 0.04 ab	14.10 ± 1.42 b	53.39 ± 3.42 b	− 0.96 ± 0.07 c	-3.63 ± 0.10 c	4.78 ± 0.07 b
Bore 3	May	11.13 ± 1.58 a	0.22 ± 0.03 a	6.41 ± 0.61 a	48.55 ± 4.31 a	− 0.63 ± 0.04 a	− 1.97 ± 0.15 a	2.93 ± 0.05 a
8.6 m	July	9.48 ± 0.95 a	0.41 ± 0.03 b	10.95 ± 0.74 b	23.96 ± 2.34 b	− 0.86 ± 0.06 b	− 2.26 ± 0.17 a	2.87 ± 0.08 a
	Sep	11.08 ± 1.52 a	0.42 ± 0.03 b	11.89 ± 0.68 b	25.96 ± 2.94 b	− 1.08 ± 0.08 c	− 1.94 ± 0.17 a	3.09 ± 0.06 a
Bore 4	May	14.34 ± 1.40 a	0.25 ± 0.03 a	6.74 ± 0.61 a	61.69 ± 5.37 a	− 0.41 ± 0.04 a	− 1.18 ± 0.04 a	2.82 ± 0.07 a
11.6 m	July	13.68 ± 1.57 a	0.38 ± 0.04 b	10.99 ± 0.85 b	35.62 ± 1.98 b	− 0.55 ± 0.03 a	− 1.95 ± 0.15 b	3.09 ± 0.09 a
	Sep	10.57 ± 1.26 a	0.22 ± 0.03 a	5.73 ± 0.59 a	52.29 ± 4.37 ab	− 0.96 ± 0.07 b	− 2.52 ± 0.23 c	2.91 ± 0.13 a
Bore 5	May	6.84 ± 0.98 a	0.14 ± 0.01 a	3.67 ± 0.36 a	49.67 ± 4.01 a	− 0.76 ± 0.05 a	− 1.66 ± 0.07 a	2.71 ± 0.03 a
14.0 m	July	13.26 ± 1.09 b	0.16 ± 0.01 a	7.45 ± 0.54 b	87.32 ± 8.69 b	− 1.28 ± 0.09 b	− 2.83 ± 0.08 b	4.68 ± 0.04 b
	Sep	10.12 ± 1.75 ab	0.24 ± 0.02 b	7.14 ± 0.42 b	40.64 ± 4.87 a	− 1.29 ± 0.04 b	− 2.74 ± 0.14 b	3.08 ± 0.05 a
Bore 6	May	15.07 ± 1.56 a	0.25 ± 0.03 a	6.28 ± 0.73 a	67.19 ± 6.30 a	− 0.46 ± 0.03 a	− 1.44 ± 0.08 a	2.61 ± 0.04 a
19.3 m	July	8.35 ± 1.50 b	0.22 ± 0.03 a	8.26 ± 0.92 a	37.20 ± 2.29 b	− 1.31 ± 0.05 b	− 2.98 ± 0.14 b	4.09 ± 0.06 b
	Sep	9.67 ± 1.64 b	0.24 ± 0.02 a	8.11 ± 0.53 a	39.70 ± 5.70 b	− 1.24 ± 0.05 b	− 1.96 ± 0.16 c	3.50 ± 0.05 b
Bore 7	May	12.13 ± 1.42 a	0.16 ± 0.02 a	4.43 ± 0.41 a	75.87 ± 4.01 a	− 0.60 ± 0.07 a	− 1.64 ± 0.15 a	2.88 ± 0.04 a
25.0 m	July	12.33 ± 1.20 a	0.17 ± 0.01 a	7.25 ± 0.53 b	72.17 ± 2.42 a	− 1.23 ± 0.08 b	− 2.79 ± 0.11 b	4.23 ± 0.07 b
	Sep	13.27 ± 1.41 a	0.32 ± 0.03 b	6.91 ± 0.43 b	41.47 ± 2.24 b	− 1.19 ± 0.07 b	− 2.51 ± 0.08 b	2.23 ± 0.03 a
Bore 8	May	10.74 ± 1.26 a	0.22 ± 0.03 a	5.47 ± 0.53 a	51.91 ± 5.19 a	− 0.43 ± 0.05 a	− 1.36 ± 0.08 a	2.56 ± 0.05 a
25.3 m	July	11.52 ± 1.52 a	0.26 ± 0.03 a	10.71 ± 0.68 b	44.52 ± 4.93 a	− 1.01 ± 0.08 b	− 3.13 ± 0.10 b	4.44 ± 0.14 b
	Sep	7.16 ± 1.33 b	0.29 ± 0.02 b	10.38 ± 0.54 b	23.77 ± 4.09 b	− 1.55 ± 0.09 c	− 3.06 ± 0.08 b	3.63 ± 0.09 c

Depth-to-groundwater (DTGW) of each site is showed as well as the significant differences (*P* < 0.05) between months in each site (different letters)

Photosynthetic rate (*A*), stomatal conductance (*g*_s_), transpiration rate (*E*), intrinsic water-use efficiency (WUEi), predawn (Ψ_pd_) and midday (Ψ_md_) water potential, and vapour pressure deficit (VPD)

**Fig. 3:**
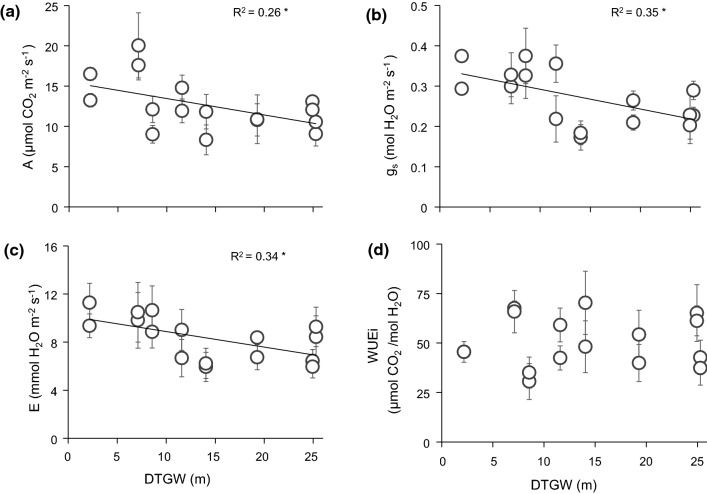
Bivariate linear regression between depth-to-groundwater (DTGW) and *Ziziphus lotus* gas-exchange rates: **a** photosynthetic rate (*A*), **b** stomatal conductance (*g*_s_), **c** transpiration rate (*E*), and **d** intrinsic water-use efficiency (WUEi). Mean values per plant are displayed ± SE. Lines represent significant linear regressions and *R*^2^, the goodness of the fit. Significance of the regression: *P < 0.05

**Fig. 4:**
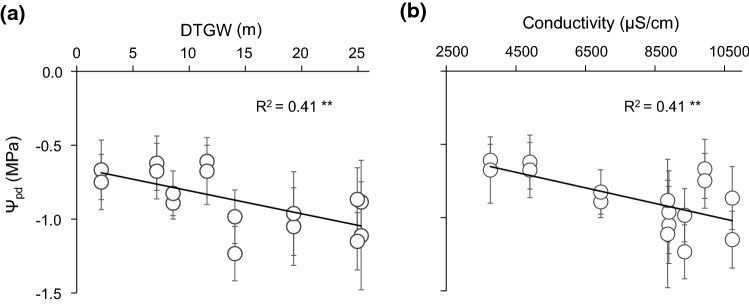
Bivariate linear regression between water potential at predawn (Ψ_pd_) of *Ziziphus lotus* and **a** depth-to-groundwater (DTGW) and **b** groundwater electrical conductivity. Mean values per plant are displayed ± SE. Lines represent significant linear regressions and *R*^2^, the goodness of the fit. Significance of the regression: **P < 0.01

**Fig. 5:**
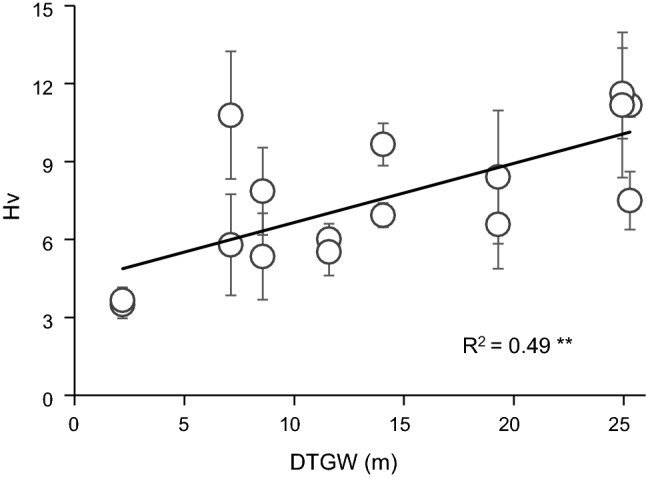
Bivariate linear regression between depth-to-groundwater (DTGW) and Huber value (Hv) of *Ziziphus lotus*. Mean values per plant are displayed ± SE. The line represents the significant linear regression and *R*^2^, the goodness of the fit. Significance of the regression: **P < 0.01

Most of the traits significantly responded to spatial (*A*, *g*_s,_ and Hv), temporal (Ψ_md_), or spatiotemporal variations (*E* and Ψ_pd_). First, bivariate linear regressions revealed a weak negative relationship with DTGW for most gas-exchange traits during the growing season (Fig. 3), except for WUEi. By contrast, no relationship was observed between these traits and groundwater salinity (Online Resource 6). However, comparing by pairs, we revealed significant differences between plants at sites with different conditions (e.g. similar DTGW and different salinity). When comparing plants at bore 4 (intermediate DTGW and low salinity) and bore 1 (low DTGW and high salinity), they only differed in *E* and Ψ_md_, showing higher water loss and also stress at bore 1 (Online Resource 7). When comparing plants from bore 4 and bore 5 (both intermediate DTGW but low and high salinity, respectively), we observed higher *E*, *g*_s_, and *A* values when salinity is lower. Regarding water potential, Ψ_pd_ was the only variable that showed a significant linear relationship to both DTGW and salinity, in which Ψ_pd_, but neither Ψ_md_ nor ΔΨ_max_, was significantly lower when DTGW and salinity were large (Fig. 4 and Online Resource 8). Nonetheless, salinity seemed to be related to more negative values of Ψ_pd_ (bore 3 and bore 5 vs. bore 4) but also Ψ_md_ (bore 5 vs. bore 4, Online Resource 7). Our results also showed that at large DTGW, plants exhibited higher Hv values than when DTGW was small (Fig. 5), even though wood density and SLA did not respond to groundwater spatial gradients (Online Resource 9). Therefore, DTGW was the main variable related to spatial variation in most single traits.

**Fig. 6:**
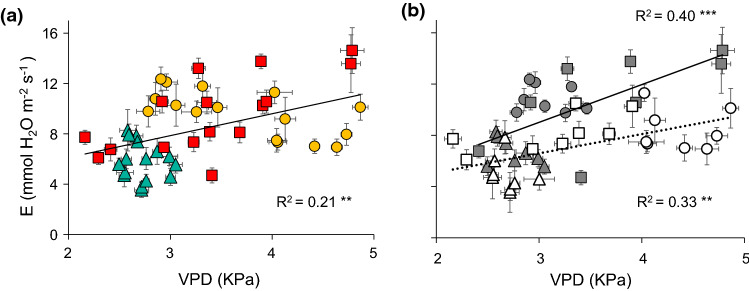
Bivariate linear regression between vapour pressure deficit (VPD) and transpiration rate (*E*) during the growing season of *Ziziphus lotus*. Mean values per plant are displayed ± SE, differentiating between **a** the three sampling periods (May: green triangles, July: yellow circles, and September: red squares), and **b** the three periods and shallow sites (DTGW < 12 m: grey symbols), and deep sites (DTGW > 12 m: open symbols). Significance of the regression: ***P < 0.001, **P < 0.01

**Fig. 7:**
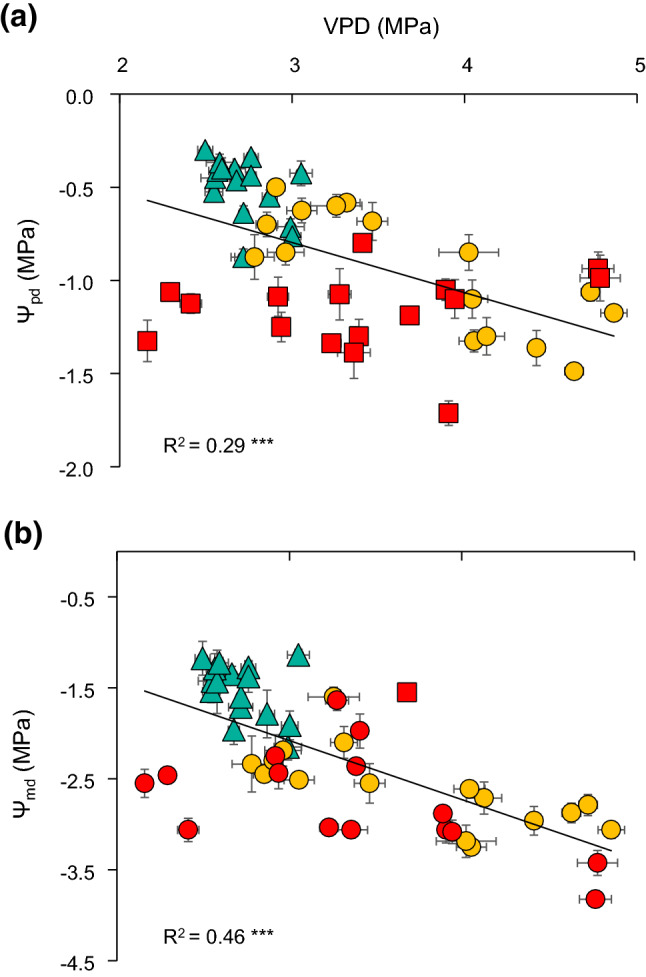
Bivariate linear regression between vapour pressure deficit (VPD) and **a** predawn, and **b** midday water potential (Ψ_pd_ and Ψ_md_, respectively**)**. Monthly values per plant are displayed ± SE. Colours and shapes represent sampling periods (May: green triangles, July: yellow circles, and September: red squares). *R*^2^ represents the goodness of the fit. Significance of the regression: ***P < 0.001

The transpiration rate was positively correlated with VPD (Fig. 6a), which represents temporal variations in climatic conditions. In May, both *E* and VPD showed lower values, with little variability across bores, whereas in summer (July and September), the increase in VPD was parallel to the rise in *E*. The general increase in VPD during the season enhanced transpiration rates more over the shallowest water tables than at the deepest ones (Fig. 6b). However, VPD did not show any significant relationship with other traits related to gas exchange (Online Resource 10). The overall increment of VPD from spring to summer was related to more negative Ψ_pd_ and Ψ_md_ values, as shown in the regression analysis (Fig. 7a, b).

Temporal analysis of the relationships between traits also revealed that *A*, *E*, and *g*_s_ were positively related to each other, despite salinity, and particularly during spring. Nonetheless, WUEi (= *A* / *g*_s_) was positively related to *A* and negatively related to *g*_s_ in summer exclusively (Online Resource 11). Our results also showed a negative relationship of Ψ_pd_ with these gas-exchange traits both in spring (*A*: *R*^2^ = 0.40, *P* = 0.008; *g*_s_: *R*^2^ = 0.37, *P* = 0.012; *E*: *R*^2^ = 0.30, *P* = 0.015) and summer (*A*: *R*^2^ = 0.25, *P* = 0.003; *g*_s_: *R*^2^ = 0.28, *P* = 0.002; *E*: *R*^2^ = 0.14, *P* = 0.037). As water availability decreased (lower Ψ_pd_), *A*, *g*_s_, and *E* were reduced, but no response was observed with an increase of plant stress (lower Ψ_md_) at any time (Online Resource 11).

### Multiple trait relationship for identifying ecophysiological thresholds

**Fig. 8:**
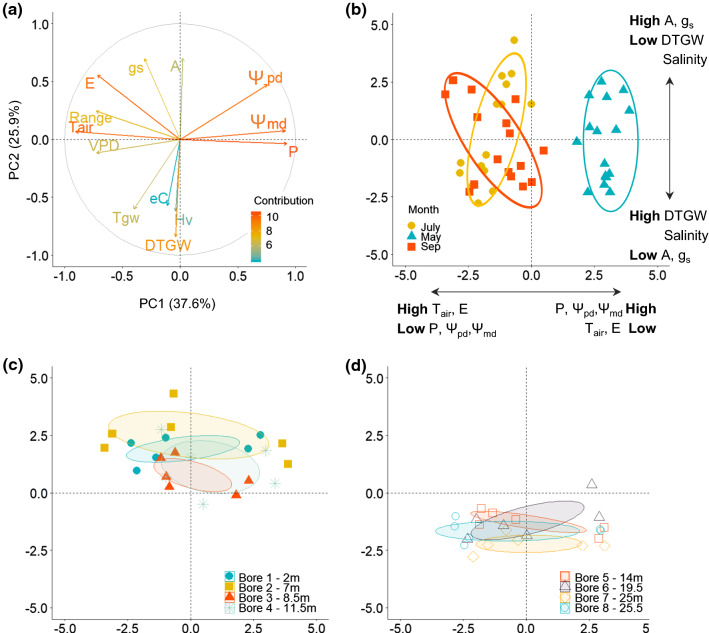
Principal component analysis (PCA). **a** Contribution of the variables from high (reddish arrows) to low contribution (bluish arrows). **b** Representation of each individual of *Ziziphus lotus* in the PCA space by month; and representation by site, differentiating between **c** sites with DTGW lower than 12 m, and **d** higher than 14 m. Horizontal and vertical arrows in panel (**b**) show the main variables contributing to each axis (PC1 and PC2, respectively): photosynthetic rate (*A*), stomatal conductance (*g*_s_), transpiration rate (*E*), predawn and midday water potential (Ψ_pd_ and Ψ_md_), maximum daily range (range), Huber value (Hv), depth-to-groundwater (DTGW), electrical conductivity (salinity, eC), vapour pressure deficit (VPD), precipitation (*P*), and air temperature (*T*_air_)

Principal component analysis (PCA) revealed multiple-trait relationships that were not identified with simple regression analysis. The two first components of the PCA explained 63.5% of the variation across plants (Fig. 8a). The first component (PC1), accounting for 37.6% of the total variation, showed strong loadings for climatic variables (i.e. *T*_air_, precipitation) as well as stem water potential (i.e. Ψ_pd_ and Ψ_md_) and *E*. The second component (PC2) explained 25.9% of the variance and showed strong loadings for groundwater traits (particularly DTGW but also *T*_GW_), *A* and *g*_s_. Groundwater salinity and Hv also contributed to PC2, although to a lesser extent. As a result, axis 1 showed a temporal gradient from the warmest and driest months that overlap to each other (July and September) with higher *E* and VPD, to the mild and humid spring (May), when water availability was higher (high Ψ_pd_) and plant stress lower (high Ψ_md_) (Fig. 8b). By contrast, axis 2 showed a DTGW gradient (Fig. 8c, d) where plants closer to the water table exhibited higher *A* and *g*_s_ but lower Hv. The PCA revealed two distinct clusters based on groundwater characteristics (DTGW and salinity) and their associated gas-exchange traits (*A*, *g*_s_): one for plants at sites with shallow DTGW (< 12 m, Fig. 8c), and the other for plants at sites with salty and deep DTGW (> 8800 µS/cm and 14 m, Fig. 8d).

## Discussion

In this study, we examined the ecophysiological response of the long-lived phreatophyte *Ziziphus lotus* to a DTGW gradient, in a coastal GDE of the Mediterranean basin. We found that DTGW and salinity had a significant, additive effect on the ecophysiological function of this phreatophyte, as hypothesised. We further found that some traits were more strongly correlated to fluctuations in DTGW and salinity (e.g. *A* and *g*_s_), whereas others were more strongly related to seasonal fluctuations in atmospheric conditions (e.g. *E*, Ψ_pd_, Ψ_md_). By applying a multiple-trait approach, we were able to identify plant ecophysiological thresholds related to the groundwater characteristics and seasonality throughout the growing season.

### Spatiotemporal variations in *Z. lotus’* traits and their relationship with groundwater

Our findings revealed spatiotemporal variations in *Z. lotus* traits, which were related to both groundwater and seasonal climatic conditions. The spatial variability in DTGW might explain the response patterns of gas exchange throughout the growing season. Increasing DTGW negatively affected carbon assimilation and water loss, as previously observed in GDEs of Australia and the United States (Butler et al. 2007; Carter and White 2009; Osuna et al. 2015; Sommer et al. 2016). Thus, deep-rooted species, particularly from arid and semiarid regions, can face physiological constraints fostered by deep water sources (Nardini et al. 2014). Here, *A*, *g*_s_, and *E* might decrease with the increase in DTGW as consequence of such constraints. In summer, the importance of groundwater availability increased, as shown by the rise in these gas-exchange rates as consequence of higher net radiation and temperature (O’Grady et al. 1999; Sommer et al. 2016), and this rise in gas-exchange rates was more pronounced in plants at shallower DTGW. On the contrary, plants at deep water tables did not experience such noticeable increase in ecophysiological activity, which could be also determined by high groundwater salinity. Even though the effects of groundwater availability and salinity cannot be uncoupled straightforwardly because of the nature of the study area, paired comparison of the sites suggested the negative effect of salinity in carbon assimilation (Online Resource 7). Nevertheless, we observed that neither carbon assimilation nor water loss was completely compromised at any point of the growing season and at any DTGW, since the lowest mean rates of *A* and *g*_s_ were observed in May, reaching 6.84 µmol CO_2_ m^−2^ s^−1^ and 0.14 mol H_2_O m^−2^ s^−1^, respectively, at bore 5 (Table 1). This result can be explained by *Z. lotus* accessing and using groundwater continuously during its growing season to avoid stomatal closure, even in summer (Torres-García et al. 2021). In this sense, the low values of WUEi we observed in summer and the lack of relationship with DTGW agree with having access to a water source, likely groundwater, since large WUEi is widely associated with groundwater usage where precipitation in scarce (Eamus et al. 2013; Cleverly et al. 2016; Rumman et al. 2018). A similar behaviour is observed in phreatophytic vegetation with access to groundwater (Nolan et al. 2017b, 2018; Rumman et al. 2018). In addition, *Z. lotus* transpiration rate did not decline in summer; in fact, it increased with VPD, more significantly at shallow water tables, suggesting that summer conditions could induce higher rates when sufficient groundwater is available (Nolan et al. 2018; Eamus and Prior, 2001), and that groundwater availability to the plant depends on climatic conditions. Despite the risk of hydraulic failure due to this anisohydric behaviour (Torres-García et al. 2021) and the physiological limitations of tapping water from deep sources, *Z. lotus* plants can maintain high gas exchange under current conditions.

The naturally occurring gradient also explained the spatial variability in Ψ_pd_ and responses to differences in water availability. Ψ_pd_ largely reflects the water potential of the rooting area (Hinckley et al. 1978) and indicates groundwater access by plants when values are barely negative (Carter and White 2009). Although *Z. lotus* plants showed values that did not fall below − 1.55 MPa, which is high given the solute potential, we found a negative trend of Ψ_pd_ not only with increasing DTGW but also with salinity. Groundwater salinity increased with DTGW away from the coast, which could be due to a marine incursion during the Holocene that penetrated the inner parts of the plain, constituting a lagoon which dried up over time and increased the salinity of the area (Vallejos et al. 2018). Therefore, it is not a recent process of seawater intrusion that induced differences in *Z. lotus* population, but a past event that fostered different salinity conditions across the landscape. This result is contrary to our assumption that seawater intrusion could affect salinity near the coast. Instead, we found that the combination of deep groundwater and high salinity away from the coast might promote water stress in the root zone as well as a drought-like condition in the plant (Kath et al. 2015). Although *Z. lotus* showed little evidence of water deficit (slightly negative water potentials even in summer) and have continuous access to groundwater during its growing season (Torres-García et al. 2021), Ψ_pd_ and Ψ_md_ correlated with DTGW and salinity. Particularly, salinity might have induced lower water potential at the root surface, reducing water uptake and photosynthetic rate in *Z. lotus*, as in other species from GDEs (Kath et al. 2015). These constraints seem to affect plants with access to intermediate and deep groundwater. In fact, a previous isotopic analysis in the area showed that *Z. lotus* plants might be reducing water uptake and triggering some stomatal regulation because of higher groundwater depth and salinity (Torres-García et al. 2021). Other authors demonstrated the accumulation of osmotically active compounds such as proline and water-soluble carbohydrates in *Z. lotus* leaves in response to salt and/or drought stress (Rais et al. 2017). Therefore, groundwater salinity might induce different adaptation mechanisms in *Z. lotus* to cope with this stress. Our results also bear consistent evidence of the salt-tolerance of *Z. lotus*, at least up to a groundwater electrical conductivity of 11000 µS/cm, and particularly at shallow groundwater tables.

Coupled with DTGW and salinity gradients, temporal groundwater depletion might induce water-deficit stress, particularly in the late-summer (Naumburg et al. 2005; Sommer et al. 2016). Our results revealed a significant decrease in both Ψ_pd_ and Ψ_md_ from spring to summer, although DTGW did not substantially decline during the growing season. We consider that the temporal fluctuations observed in groundwater level are insufficient to induce such a response, as maximum differences reported during the growing season reached just 18 cm in bore 8. Even daily fluctuations observed at the shallowest and closer-to-the-coast sites, which can reflect groundwater use due to transpiration (Dahm et al. 2002; Thibault et al. 2017) or the effect of tides on the coastal aquifer (Vallejos et al. 2015; Levanon et al. 2017), had little effect on groundwater salinity. In addition, the slight differences observed in groundwater temperature are insufficient to substantive affect viscosity related to DTGW or xylem water assent and thus, to infer large physiology effect on vegetation (Jensen and Taylor, 1961). Thus, the significant decrease in the water potential during the growing season was due to other factors such as atmospheric evaporative demand. The negative response of both Ψ_pd_ and Ψ_md_ to increased VPD throughout the growing season shows the decisive effect of the high summer temperature on plant regulation, highlighting the importance of VPD in promoting transpiration when water availability is not limiting (Sulman et al. 2016; Amitrano et al. 2019). However, in these GDEs where daily and seasonal groundwater fluctuations are minor, phreatophytes run the risk of maximising productivity over safety (Hultine et al. 2020), which can also be fostered by the anisohydric behaviour of the species (Torres-García et al. 2021). Being an anisohydric phreatophyte in arid and semiarid regions seems to be a risky option, which can only be overcome in some species by plasticity in individuals for responding to upcoming environmental conditions through shifts in hydraulic traits such us higher root area to leaf area ratios or higher resistance to xylem cavitation (Hultine et al. 2020).

Different responses observed in *Z. lotus* transpiration rates could also be generated by differences in xylem traits such as sapwood area (Attia et al. 2015), or in leaf area. Our results revealed that Hv (the ratio of sapwood area to leaf area) was higher at deeper groundwater sites, as already reported for other phreatophytes of mesic (Zolfaghar et al. 2014) and xeric environments (Carter and White 2009). Larger Hv is observed in drought-tolerant plants (Canham et al. 2009) because of higher sapwood area to support leaf area and/or less leaf area supported by such sapwood (Carter and White 2009). Higher sapwood area observed in plants at deep sites (Online Resource 12) could enhance *Z. lotus* capacity for water supply (Butterfield et al. 2021), and compensate the evaporative demand, particularly in summer. On the one hand, *Z. lotus* plants with less reliable groundwater supply (deep DTGW) seem to make smaller investments in leaf area than plants at shallow sites (Online Resource 12). This mechanism might allow *Z. lotus* to cope with reduced water availability by decreasing their hydraulic demand, and therefore, their transpiration rates at a canopy level (Gazal et al. 2006; Carter and White 2009; Zolfaghar et al. 2014). Indeed, reductions of aboveground biomass are acknowledged to be a common adaptation when plants cannot overcome the anatomical and functional adaptation cost of water scarcity (Naumburg et al. 2005).

In contrast to Hv, wood density was largely independent of groundwater because it depends on development of modified cell types (e.g. xylem vessels, fibres) (Lachenbruch and McCulloh 2014). Likewise, our results showed that SLA was independent of DTGW, as has been reflected in some studies along water availability gradients (Nolan et al. 2017a). In this case, as SLA refers to the ratio of leaf area to leaf dry mass, or the inverse of leaf thickness (Pérez-Harguindeguy et al. 2013), SLA would be conserved as an adaptation to light levels and aridity. By contrast, leaf area reductions are medium-to-long-term adaptations to limit water loss (Zolfaghar et al. 2014) that *Z. lotus* might have developed to address DTGW coupled to weak stomatal control (i.e. anisohydry). Despite being able to explain the variability of plant traits, the weak but significant relationships obtained revealed how difficult it is to define the functioning of a complex ecosystem like a GDE by a single regression for a given pair of traits.

### Ecophysiological thresholds and future considerations

Assessing the expression of multiple traits provides tools to predict patterns of change in GDEs in response to variability in groundwater and across seasons (Hultine et al. 2020). A multiple-trait analysis revealed that the variability observed in the functioning of *Z. lotus* could be explained by the combination of both temporal variations in climatic conditions during the growing season of the species and the spatial differences in groundwater characteristics of the study area. Temporal differences from spring to summer showed a decrease in water potential with increased transpiration rates, promoted by environmental conditions (lower humidity, higher temperatures, and evaporative demand). This response could have fostered evaporative cooling, regulating leaf temperature for maintaining the plant carbon balance (Drake et al. 2018) and suggesting the decline in water potential was insufficient to indicate water stress. Thus, *Z. lotus* plants could avoid extreme thermal stress that can damage the photosynthetic machinery whilst preventing a steep decline in photosynthetic rate. However, sufficient water availability is required to maintain evaporative cooling, which is essential under ongoing increases of both mean air temperatures and the severity of heat waves (Urban et al. 2017).

By contrast to temporal fluctuations, the ecophysiological functioning of *Z. lotus* across space was explained by the combination of groundwater availability (mainly determined by DTGW) and salinity (expressed by electrical conductivity). Salinity is commonly present in arid ecosystems with phreatophytic vegetation because of reduced precipitation, which prevents leaching of salts, and evaporation, which leaves salts behind (Glenn et al. 2013). We found that the DTGW gradient coincided with a salinity gradient such that the deepest groundwater was also saltiest. Without the ability to discriminate between these characteristics at high depths, we observed that higher groundwater salinity combined with larger DTGW affected the ecophysiology of *Z. lotus* and promoted remarkable differences along the naturally occurring gradient. Notwithstanding, at shallow sites, the effect of salinity is blurred by greater water availability, suggesting that DTGW is a more determinant factor for *Z. lotus* ecophysiological functioning. Thus, we identified a response threshold at 12–14 m, mainly promoted by differences in gas-exchange rates, which is consistent with previous studies about the species (Torres-García et al. 2021). Saltier and deeper groundwater have a substantial effect on plants, reducing water uptake, and diminishing gas exchange (Kath et al. 2015). Such threshold might point to the DTGW limits for maintaining high ecophysiological functioning and could be used as a baseline for managing this GDE.

Under predicted climate change for semiarid regions of the Mediterranean basin, anisohydric phreatophytes like *Z. lotus* would increase their transpiration rates as well as the risk of hydraulic failure despite their relative drought tolerance (McDowell et al. 2008). For the related GDE, this means that an increase in groundwater discharge and associated increases in DTGW could also promote salinization (Jobbágy and Jackson 2007; Runyan and D’Odorico 2010). The expected decrease in precipitation will not support recharge or salt leaching, and salinization can continue until it reaches the tolerance threshold of the species. Once salinity intolerance is reached, further groundwater uptake might be compromised, along with plant survival (Nosetto et al. 2008). Furthermore, processes of seawater intrusion can occur in coastal aquifers because of the reduction in groundwater, what would result in ecosystem-scale changes in hydraulic and functional traits (Runyan and D’Odorico 2010; Hultine et al. 2020). The concern is also whether a depletion in groundwater level would exceed the root growth rate (Orellana et al. 2012), or even if temporal fluctuations would have a long-term impact on plant ecophysiology. In the case of the long-lived phreatophyte *Z. lotus*, our results suggest that its salt-tolerance confers to the plants the ability to escape from the effect of the stress when groundwater availability is greater. However, phreatophytes that obtain groundwater from deep water tables and that already experience some physiological constraints (e.g. over 14 m in the case of *Z. lotus*), could be intensively jeopardised by groundwater variations in the future.

## Conclusions

In this research, we assessed spatiotemporal variations both in groundwater properties of a GDE in a semiarid region and in the morpho-functional traits of the phreatophyte that dominates this ecosystem: *Ziziphus lotus*. The naturally occurring DTGW gradient and associated monitoring field station have provided an interesting scenario to assess ecophysiological differences related to water availability for phreatophytic vegetation. Here, we show that both groundwater depth and salinity are highly connected to the ecophysiological functioning of phreatophytic vegetation in drylands. Nevertheless, no evidence of seawater intrusion seemed to affect *Z. lotus* plants so far, and groundwater salinity could be related to past events of seawater rise. Differences in climatic conditions throughout the growing season drove temporal variability in *Z. lotus* response, with summer conditions promoting carbon assimilation and water loss in this winter-deciduous phreatophyte, more intensively at shallow water tables. The multiple-trait analysis led to identifying spatial and temporal ecophysiological thresholds that depend on groundwater availability and salinity, as well as atmospheric evaporative demand. Under the expected reductions in groundwater reservoirs as consequence of both climate aridification and the increase in groundwater consumption and drawdown by human overexploitation, understanding the functioning of GDEs of arid and semiarid regions and defining ecophysiological thresholds of their phreatophytic vegetation will provide valuable insight to face upcoming management challenges.

## Supplementary Information

Below is the link to the electronic supplementary material.

Supplementary file1 (DOCX 30 KB)

Supplementary file2 (DOCX 19 KB)

Supplementary file3 (DOCX 967 KB)

Supplementary file4 (DOCX 17 KB)

Supplementary file5 (DOCX 17 KB)

Supplementary file6 (DOCX 57 KB)

Supplementary file7 (DOCX 106 KB)

Supplementary file8 (DOCX 58 KB)

Supplementary file9 (DOCX 58 KB)

Supplementary file10 (DOCX 75 KB)

Supplementary file11 (DOCX 255 KB)

Supplementary file12 (DOCX 27 KB)

## Data Availability

The dataset used during the current study is available from the corresponding author on reasonable request.
