# Supplementary material for: A multiple-trait analysis of ecohydrological acclimatisation in a dryland phreatophytic shrub
Source: Oecologia. 2021 Jul 31;196(4):1179–93. doi: 10.1007/s00442-021-04993-w (PMC8367881; doi:10.1007/s00442-021-04993-w)
Supplement: Supplementary file 1 — Supplementary file1 (DOCX 30 KB) [file 442_2021_4993_MOESM1_ESM.docx]

**Online resource 1.** Meteorological data from Almería airport meteorological station (Spanish meteorological agency) located 8 km from the study area. Monthly precipitation (P) and mean monthly temperature (Tair) from 2019 are shown as well as mean precipitation and temperature from the period comprised between 2000 and 2020.
