# Supplementary material for: A multiple-trait analysis of ecohydrological acclimatisation in a dryland phreatophytic shrub
Source: Oecologia. 2021 Jul 31;196(4):1179–93. doi: 10.1007/s00442-021-04993-w (PMC8367881; doi:10.1007/s00442-021-04993-w)
Supplement: Supplementary file 2 — Supplementary file2 (DOCX 19 KB) [file 442_2021_4993_MOESM2_ESM.docx]

**Online resource 2.** Monthly mean depth-to-groundwater (DTGW), electrical conductivity, and groundwater temperature (T_gw_) ± standard deviation of the study area. Letters show significant differences between months in each bore (P < 0.05) from the one-way ANOVAs of the log-transformed data.

| Site | Month | DTGW (m) | Conductivity (µS/cm) | | T_GW_ (ºC) |
| --- | --- | --- | --- | --- | --- |
| Bore 1 | May | 2.216 ± 0.062 a | | 9886.94 ± 232.68 a | 22.34 ± 0.16 a |
|  | June | 2.204 ± 0.039 b | | 9724.87 ± 356.32 b | 22.76 ± 0.14 b |
|  | July | 2.180 ± 0.031 c | | 9931.36 ± 144.60 c | 23.24 ± 0.40 c |
|  | August | 2.211 ± 0.027 d | | 10238.92 ± 172.30 d | 23.48 ± 0.44 d |
|  | September | 2.219 ± 0.036 e | | 10558.87 ± 79.92 e | 23.44 ± 0.36 e |
| Bore 2 | May | 7.540 ± 0.107 a | | 4999.83 ± 136.73 a | 21.78 ± 0.09 a |
|  | June | 7.393 ± 0.220 b | | 4923.47 ± 116.03 b | 21.87 ± 0.07 b |
|  | July | 7.111 ± 0.028 c | | 4901.45 ± 98.18 c | 22.05 ± 0.08 c |
|  | August | 7.144 ± 0.018 d | | 4887.18 ± 56.33 d | 22.36 ± 0.11 d |
|  | September | 7.175 ± 0.015 e | | 4915.15 ± 37.81 c | 22.60 ± 0.03 e |
| Bore 3 | May | 8.568 ± 0.038 ab | | 6727.07 ± 236.24 a | 23.20 ± 0.02 a |
|  | June | 8.575 ± 0.022 c | | 6882.98 ± 162.60 b | 23.33 ± 0.05 b |
|  | July | 8.562 ± 0.021 a | | 6941.25 ± 158.59 c | 23.45 ± 0.04 c |
|  | August | 8.569 ± 0.015 b | | 6947.63 ± 227.98 c | 23.50 ± 0.04 d |
|  | September | 8.574 ± 0.020 bc | | 7067.78 ± 218.15 d | 23.54 ± 0.03 e |
| Bore 4 | May | 11.570 ± 0.012 a | | 3505.97 ± 91.94 a | 23.08 ± 0.03 a |
|  | June | 11.578 ± 0.010 ab | | 3594.25 ± 137.55 b | 23.24 ± 0.07 b |
|  | July | 11.579 ± 0.007 b | | 3763.78 ± 118.79 c | 23.41 ± 0.04 c |
|  | August | 11.603 ± 0.015 c | | 3638.82 ± 110.58 d | 23.49 ± 0.01 d |
|  | September | 11.632 ± 0.007 d | | 3489.15 ± 24.97 e | 23.53 ± 0.02 e |
| Bore 5 | May | 14.029 ± 0.011 a | | 10066.35 ± 79.38 a | 22.79 ± 0.03 a |
|  | June | 14.038 ± 0.010 ab | | 9924.46 ± 253.57 b | 22.93 ± 0.05 b |
|  | July | 14.050 ± 0.008 c | | 9348.57 ± 138.00 c | 23.05 ± 0.03 c |
|  | August | 14.040 ± 0.008 b | | 9576.74 ± 51.84 d | 23.12 ± 0.01 d |
|  | September | 14.016 ± 0.007 d | | 9604.76 ± 5.43 e | 23.14 ± 0.01 e |
| Bore 6 | May | 19.249 ± 0.012 a | | 8850.21 ± 9.61 ab | 23.38 ± 0.02 a |
|  | June | 19.274 ± 0.023 b | | 8848.58 ± 27.72 a | 23.51 ± 0.05 b |
|  | July | 19.284 ± 0.010 b | | 8896.21 ± 86.76 c | 23.65 ± 0.04 c |
|  | August | 19.282 ± 0.009 b | | 8865.31 ± 6.29 b | 23.74 ± 0.03 d |
|  | September | 19.291 ± 0.007 b | | 8885.51 ± 11.90 c | 23.81 ± 0.02 e |
| Bore 7 | May | 24.905 ± 0.036 a | | 10178.71 ± 530.56 a | 23.30 ± 0.03 a |
|  | June | 24.929 ± 0.025 b | | 10363.50 ± 192.98 b | 23.45 ± 0.05 b |
|  | July | 24.942 ± 0.014 b | | 10718.59 ± 72.17 c | 23.57 ± 0.03 c |
|  | August | 24.966 ± 0.017 c | | 10931.73 ± 46.89 d | 23.65 ± 0.03 d |
|  | September | 24.976 ± 0.016 c | | 11026.51 ± 11.72 e | 23.72 ± 0.01 e |
| Bore 8 | May | 25.231 ± 0.009 a | | 8944.37 ± 3.98 a | 23.51 ± 0.03 a |
|  | June | 25.238 ± 0.023 a | | 8906.29 ± 20.50 b | 23.66 ± 0.06 b |
|  | July | 25.286 ± 0.021 b | | 8862.76 ± 21.27 c | 23.81 ± 0.04 c |
|  | August | 25.364 ± 0.023 c | | 8822.75 ± 9.59 d | 23.91 ± 0.03 d |
|  | September | 25.408 ± 0.008 d | | 8796.77 ± 7.24 e | 23.98 ± 0.02 e |
