# Supplementary material for: A multiple-trait analysis of ecohydrological acclimatisation in a dryland phreatophytic shrub
Source: Oecologia. 2021 Jul 31;196(4):1179–93. doi: 10.1007/s00442-021-04993-w (PMC8367881; doi:10.1007/s00442-021-04993-w)
Supplement: Supplementary file 3 — Supplementary file3 (DOCX 967 KB) [file 442_2021_4993_MOESM3_ESM.docx]

**Online resource 3.** Spatiotemporal variations in groundwater temperature, T_GW_ (a) and groundwater electrical conductivity (b) for the study period (15 May 2019 - 11 September 2019).
