# Supplementary material for: A multiple-trait analysis of ecohydrological acclimatisation in a dryland phreatophytic shrub
Source: Oecologia. 2021 Jul 31;196(4):1179–93. doi: 10.1007/s00442-021-04993-w (PMC8367881; doi:10.1007/s00442-021-04993-w)
Supplement: Supplementary file 4 — Supplementary file4 (DOCX 17 KB) [file 442_2021_4993_MOESM4_ESM.docx]

**Online resource 4**. Results of the two-way ANOVA applied to the morpho-functional and hydraulic variables of *Ziziphus lotus*: photosynthetic rate (A), stomatal conductance (g_s_), transpriation rate (E), intrinsic water-use efficiency (WUEi), predawn (Ψ_pd_) and midday (Ψ_md_) water potential, and vapour pressure defficit (VPD). Significance and degree of freedom of the sum of squares of each trait are shown. Significance: ****P* < 0.001, ***P* < 0.01, n.s: no significance.

|  |  | A | g_s_ | E | WUEi | Ψ_pd_ | Ψ_md_ | VPD |
| --- | --- | --- | --- | --- | --- | --- | --- | --- |
| Bore | df = 7 | *** | *** | *** | *** | *** | *** | ** |
| Month | df = 2 | n.s | *** | *** | *** | *** | *** | *** |
| Interaction | df = 14 | *** | *** | *** | *** | *** | *** | *** |
