# Supplementary material for: A multiple-trait analysis of ecohydrological acclimatisation in a dryland phreatophytic shrub
Source: Oecologia. 2021 Jul 31;196(4):1179–93. doi: 10.1007/s00442-021-04993-w (PMC8367881; doi:10.1007/s00442-021-04993-w)
Supplement: Supplementary file 5 — Supplementary file5 (DOCX 17 KB) [file 442_2021_4993_MOESM5_ESM.docx]

**Online resource 5.** Mean Huber value (Hv), Specific Leaf Area (SLA), and wood density ( ± standard error) of *Ziziphus lotus* at each site. Letters show significant difference between sites (P < 0.05) from one-way ANOVA.

| Bores | Hv | SLA (cm^2^/g) | Wood density (g/cm^3^) |
| --- | --- | --- | --- |
| Bore 1 (2.2 m) | 3.58 ± 0.08 a | 86.33 ± 7.01 a | 0.82 ± 0.02 a |
| Bore 2 (7.3 m) | 8.29 ± 2.49 ab | 99.04 ± 9.15 a | 0.79 ± 0.02 a |
| Bore 3 (8.6 m) | 6.60 ± 1.26 ab | 70.37 ± 3.71 a | 0.78 ± 0.01 a |
| Bore 4 (11.6 m) | 5.77 ± 0.25 ab | 79.73 ± 6.15 a | 0.74 ± 0.08 a |
| Bore 5 (14.0 m) | 8.30 ± 1.36 ab | 78.58 ± 4.98 a | 0.79 ± 0.07 a |
| Bore 6 (19.3 m) | 7.50 ± 0.91 ab | 87.16 ± 8.17 a | 0.80 ± 0.02 a |
| Bore 7 (25.0 m) | 11.40 ± 0.22 b | 82.83 ± 5.31 a | 0.76 ± 0.03 a |
| Bore 8 (25.3 m) | 9.34 ± 0.84 b | 85.75 ± 7.51 a | 0.85 ± 0.05 a |
