# Supplementary material for: A multiple-trait analysis of ecohydrological acclimatisation in a dryland phreatophytic shrub
Source: Oecologia. 2021 Jul 31;196(4):1179–93. doi: 10.1007/s00442-021-04993-w (PMC8367881; doi:10.1007/s00442-021-04993-w)
Supplement: Supplementary file 6 — Supplementary file6 (DOCX 57 KB) [file 442_2021_4993_MOESM6_ESM.docx]

**Online resource 6.** Bivariate linear regression between gas-exchange traits of *Ziziphus lotus* (photosynthetic rate, A; stomatal conductance, g_s_; transpriation rate, E; and intrinsic water use efficiency, WUEi) and groundwater conductivity (a, c, e, g) and groundwater temperature (b, d, f, h). Mean values per plant are displayed ± standard error. Lines represent the linear regression, *R*^2^, the goodness of the fit, and *P*, the significance of each analysis (no data: no significance).
