# Supplementary material for: A multiple-trait analysis of ecohydrological acclimatisation in a dryland phreatophytic shrub
Source: Oecologia. 2021 Jul 31;196(4):1179–93. doi: 10.1007/s00442-021-04993-w (PMC8367881; doi:10.1007/s00442-021-04993-w)

**Online resource 7.** Paired comparison of leaf gas exchange traits and water potential of *Ziziphus lotus* plants at sites with different groundwater characteristics. The number of wavy lines bellow each number (bore number) represents depht-to-groundwater, from shallow (4 lines) to deep groundwater (1 line); colors indicate levels of salinity: hight (red), intermediate (yellow), low (blue). Mean values per site are indicated ± standard error. E: transpriation rate; gs: stomatal conductance; A: photosynthetic rate; Ψ_md_: midday water potential; Ψ_pd_: predawn water potential. Note that only significant differences between these bores from each variable are included (t-test, P < 0.05).


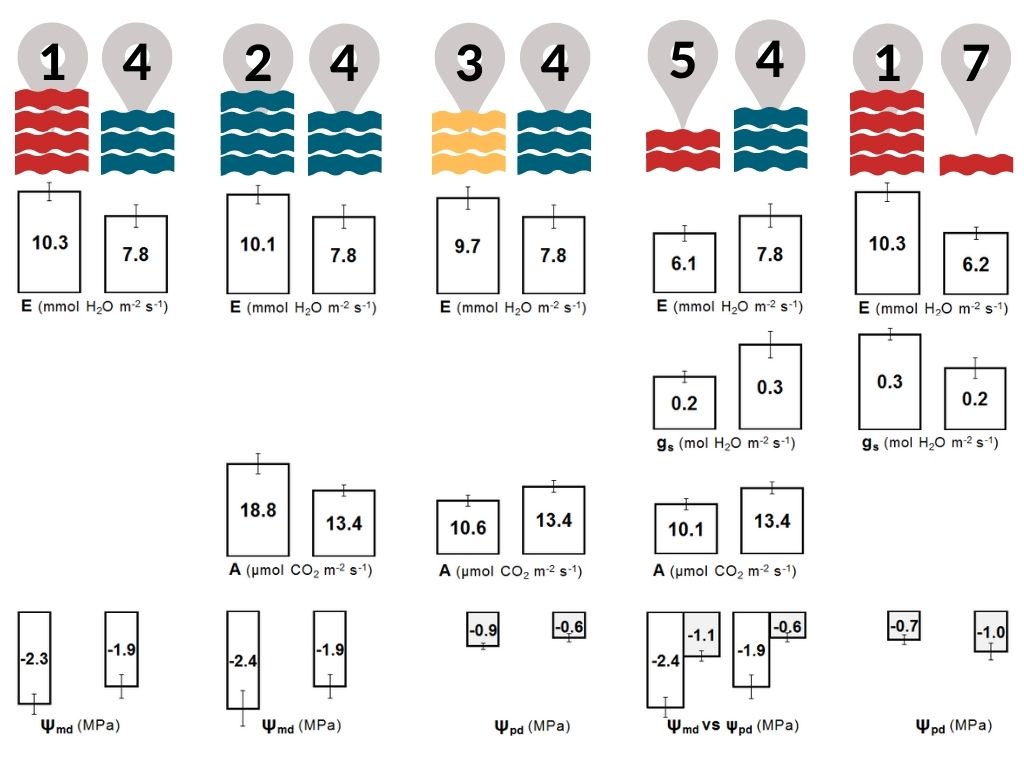

Supplement: Supplementary file 7 — Supplementary file7 (DOCX 106 KB) [file 442_2021_4993_MOESM7_ESM.docx]
