# Supplementary material for: A multiple-trait analysis of ecohydrological acclimatisation in a dryland phreatophytic shrub
Source: Oecologia. 2021 Jul 31;196(4):1179–93. doi: 10.1007/s00442-021-04993-w (PMC8367881; doi:10.1007/s00442-021-04993-w)
Supplement: Supplementary file 8 — Supplementary file8 (DOCX 58 KB) [file 442_2021_4993_MOESM8_ESM.docx]

**Online resource 8.** Bivariate linear regression between groundwater characteristics (depth-to-groundwater, electrical conductivity, temperature) and hydraulic traits (Ψ_pd_: predawn water potential, Ψ_md_: midday water potential, ΔΨ_max_: maximum daily range). Mean values per plant are displayed ± standard error. Lines represent the linear regression, *R*^2^, the goodness of the fit, and *P*, the significance of each analysis (no data: no significance).
