# Supplementary material for: A multiple-trait analysis of ecohydrological acclimatisation in a dryland phreatophytic shrub
Source: Oecologia. 2021 Jul 31;196(4):1179–93. doi: 10.1007/s00442-021-04993-w (PMC8367881; doi:10.1007/s00442-021-04993-w)
Supplement: Supplementary file 9 — Supplementary file9 (DOCX 58 KB) [file 442_2021_4993_MOESM9_ESM.docx]

**Online resource 9.** Bivariate linear regression between groundwater characteristics and morphological traits: Huber value (Hv), Wood density, and Specific leaf area (SLA). Mean values per plant are displayed ± standard error. Lines represent the linear regression, *R*^2^, the goodness of the fit, and *P*, the significance of each analysis (no data: no significance).
