# Supplementary material for: A multiple-trait analysis of ecohydrological acclimatisation in a dryland phreatophytic shrub
Source: Oecologia. 2021 Jul 31;196(4):1179–93. doi: 10.1007/s00442-021-04993-w (PMC8367881; doi:10.1007/s00442-021-04993-w)
Supplement: Supplementary file 10 — Supplementary file10 (DOCX 75 KB) [file 442_2021_4993_MOESM10_ESM.docx]

**Online resource 10**. Relationship between vapour pressure deficit (VPD) and gas-exchange traits: photosynthetic rate (A), stomatal conductance (g_s_), and intrinsic water-use efficiency (WUEi) (nonsignificant). Monthly values per plant are displayed ± standard error. Colours and shapes represent sampling periods (May: green triangles, July: yellow circles, and September; red squares).
