# Supplementary material for: A multiple-trait analysis of ecohydrological acclimatisation in a dryland phreatophytic shrub
Source: Oecologia. 2021 Jul 31;196(4):1179–93. doi: 10.1007/s00442-021-04993-w (PMC8367881; doi:10.1007/s00442-021-04993-w)
Supplement: Supplementary file 11 — Supplementary file11 (DOCX 255 KB) [file 442_2021_4993_MOESM11_ESM.docx]

**Online resource 11.** Bivariate linear regression between gas-exchange and hydraulic traits of *Ziziphus lotus*: photosynthetic rate (A), stomatal conductance (g_s_), transpriation rate (E), intrinsic water-use efficiency (WUEi), and predawn (Ψ_pd_) and midday (Ψ_md_) water potential. Montly values per plant are displayed ± standard error. Colours and shapes represent sampling periods (May: green triangles, July: yellow circles, and September; red squares). Lines represent significant linear regression: dotted lines and boxes show spring regression analysis (May) and solid lines and boxes represent summer regression (July + September). The goodness of the fit (*R*^2^) and the significance of each analysis (*P*) are showed in the respective boxes.
